# A tale of two neglected tropical infections: using GIS to assess the spatial and temporal overlap of schistosomiasis and leprosy in a region of Minas Gerais, Brazil

**DOI:** 10.1590/0074-02760160395

**Published:** 2017-04

**Authors:** David Alexander Phillips, José Antonio Ferreira, Deidra Ansah, Herica SA Teixeira, Uriel Kitron, Thelma de Filippis, Marcelo H de Alcântara, Jessica K Fairley

**Affiliations:** 1 Augusta University/University of Georgia Medical Partnership, Athens, GA, United States; University of Georgia, University of Georgia, Athens, GA, United States; 2 Escola de Medicina, Faculdade da Saúde e Ecologia Humana, Vespasiano, MG, Brasil; 3 Emory University School of Medicine, Department of Pediatrics, Atlanta, GA, United States; 4 Secretaria Municipal de Saúde, Vespasiano, MG, Brasil; 5 Emory University, Department of Environmental Science, Atlanta, GA, United States; 6 Emory University School of Medicine, Department of Medicine, Division of Infectious Diseases, Atlanta, GA, United States

**Keywords:** leprosy, schistosomiasis, coinfection, geographic, information systems, Brazil

## Abstract

**BACKGROUND:**

Despite public health efforts to reduce the global burden of leprosy, gaps remain in the knowledge surrounding transmission of infection. Helminth co-infections have been associated with a shift towards the lepromatous end of the disease spectrum, potentially increasing transmission in co-endemic areas.

**OBJECTIVES:**

Using this biologically plausible association, we conducted a geographic information systems (GIS) study to investigate the spatial associations of schistosomiasis and leprosy in an endemic area of Minas Gerais (MG), Brazil.

**METHODS:**

Data on new cases of *Mycobacterium leprae* and *Schistosoma mansoni* infections from 2007-2014 were retrieved from the Brazilian national notifiable diseases information system for seven municipalities in and surrounding Vespasiano, MG. A total of 139 cases of leprosy and 200 cases of schistosomiasis were mapped to a municipality level. For one municipality, cases were mapped to a neighborhood level and a stratified analysis was conducted to identify spatial associations.

**FINDINGS:**

A relative risk of 6.80 [95% confidence interval (CI) 1.46 - 31.64] of leprosy was found in neighborhoods with schistosomiasis. Incidence rates of leprosy increased with corresponding incidence rates of schistosomiasis, and the temporal trends of both infections were similar.

**CONCLUSIONS:**

The associations found in this project support the hypothesis that helminth infections may influence the transmission of leprosy in co-endemic areas.

Despite public health interventions and the availability of multidrug therapy, leprosy has continued to affect communities at a steady rate since 2005 with an average of about 200,000 new cases annually (Rodrigues & Lockwood 2011, GL 2012, White & Franco-Paredes 2015). While transmission is thought to be predominantly human-to-human through respiratory droplets, the inability to culture the bacteria in vitro, the lack of an effective animal model (only armadillos), and a long incubation period have limited studies on disease transmission (Rodrigues & Lockwood 2011). Passive and active surveillance have failed to significantly impact disease control over the last ten years (Smith et al. 2014). Therefore, new strategies to understand and reduce transmission of *Mycobacterium leprae* are critically needed.

*M. leprae* causes a complex infection with a spectrum of different clinical manifestations and immunologic responses (White & Franco-Paredes 2015). Multibacillary (MB) leprosy, especially lepromatous disease, is notable for a lack of a strong Th1 response and the presence of Th2 associated cytokines and inflammatory markers (Nath et al. 2015). These MB cases are believed to be the source of infections to other susceptible individuals, while paucibacillary cases (PB) have a characteristic robust Th1 response and are thought to be less infectious (Nath et al. 2015). Why some patients get PB and some get MB disease is not well understood but thought to be related to host susceptibility factors, such as genetic causes (Sapkota et al. 2010, White & Franco-Paredes 2015, Schreuder et al. 2016). Poverty is also associated with infection, but how it facilitates transmission is not completely understood. Proposed factors include overcrowding, contaminated soil, and undernutrition (Feenstra et al. 2011, Cabral-Miranda et al. 2014, Freitas et al. 2014). Helminth infections are predominantly diseases of poverty and affect close to 2 billion people worldwide (WHO 2016a, b). Furthermore, helminth co-infections have been shown to be associated with MB disease in small studies, with the hypothesis that the chronic immune activation of the Th2 response by helminths makes the individual more likely to have MB disease (Diniz et al. 2001, 2010), 2010). Therefore, this could increase the infectious reservoir in the community when co-infections are present. Studying the spatial overlap of select helminth infections and leprosy, therefore, could lend support to this hypothesis and support further studies and expand control strategies. In Brazil, coordinated efforts to address both helminth infections and leprosy occurred in 2013 as part of the Ministry’s of Health’s “Integrated Strategic Action Plan for elimination of leprosy, lymphatic filariasis, onchocerciasis, schistosomiasis, trachoma as a cause of blindness and control of soil-transmitted helminthiases (STH)”. This program combines targeted screening for leprosy with mass drug administration campaigns in school-aged children. Therefore, identifying spatial associations can lend more support to the expansion and development of similar programs in Brazil and other endemic areas.

Geographic information systems (GIS) coupled with spatial analysis is a rapidly growing field and has become an important tool to study disease epidemiology. Spatial analysis can detect clusters of disease that aggregate data can miss, thus making it very useful to study the transmission of infections (Clennon et al. 2004, Fonseca et al. 2014). Furthermore, it can take into account environmental factors that may influence disease incidence (Fonseca et al. 2014). GIS has been useful to study both Hansen’s disease and schistosomiasis in Brazil and in other endemic areas (Sampaio et al. 2013, Barreto et al. 2014, Fonseca et al. 2014, Lai et al. 2015, Monteiro et al. 2015). It has helped increase new case detection rates of leprosy in a hyperendemic area in Northern Brazil by allowing for targeted interventions in areas of clustering (Barreto et al. 2015). Therefore, given the biologically plausible hypothesis that leprosy-helminth co-infections could increase the reservoir of *M. leprae* infection, it is an ideal mode to study the relationship and spatial overlap of these two infections. Our study presents the first known analysis of the spatial and temporal overlap of leprosy and schistosomiasis in a region of Minas Gerais (MG) state, Brazil.

## MATERIALS AND METHODS

*Study area* - Municipality data from seven municipalities surrounding Vespasiano, MG, Brazil, were included in this study. These included Vespasiano, Confins, Matozinhos, Pedro Leopoldo, Santana do Riacho, Lagoa Santa and São José da Lapa. This area is endemic with leprosy, *Schistosoma mansoni* infection and visceral leishmaniasis (VL). For the neighborhood level analysis, the most populous municipality, Vespasiano, was used due to the availability of neighborhood population data. There were 44 neighborhoods in Vespasiano for which data were available. In Vespasiano, there are minimal natural water sources that could transmit schistosomiasis and *S. mansoni* was not found in snails in this municipality in a prior investigation (Souza et al. 1998). Therefore, most residents are believed to have contracted the infection elsewhere, likely in nearby municipalities in MG.

*GIS mapping* - Data on new cases of leprosy, schistosomiasis and VL, all compulsory reportable diseases in Brazil, were retrieved from the national notifiable diseases information system (SINAN) for the years 2007-2014 for the seven municipalities described above. Data on VL were included to compare schistosomiasis against another endemic disease associated with poverty but not hypothesized to be associated with increased leprosy transmissibility. The de-identified data collected included age at diagnosis, sex, municipality and neighborhood of residence, class of leprosy disease (MB vs. PB), and date of diagnosis. For all three diseases, the cases were first mapped to the municipality level with ArcGIS (v10.3.1) using publicly available maps of MG [Instituto Brasileiro de Geografia e Estatística (http://www.ibge.gov.br/)]. The cases of leprosy and schistosomiasis were then mapped to the neighborhood level (0.5-1 km^2^ on average) for Vespasiano. Census tract data on cases were not available making neighborhoods the smallest geospatial units available for this study. VL cases were mapped to the municipality level for general incidence rates and to analyse the temporal pattern of infections. The VL cases did not have complete neighborhood level data and were not included in the Vespasiano analysis. A list of official neighborhoods with corresponding population data from 2014 was provided by the Vespasiano Secretary of Health. The boundaries of neighborhoods were determined using maps provided by municipalities where available. In areas without municipal maps, neighborhood boundaries were determined by comparing crowd-sourced maps (Wikimapia and OpenStreetMap) with at least two private local real estate companies, as well as with publicly available urban census tracts. Neighborhoods were drawn in ArcGIS when the real estate data corroborated the crowd-sourced maps and these outlines matched those of one or a combination of census tracts. While different sources had slightly different numbers and names of neighborhoods, the 44 used in this study corresponded best with the reported case data from SINAN and the population data from the secretary of health. These maps were also overlaid with 2014 purchasing power per capita data made available through Esri’s ArcGIS map service, which allowed for a comparison of one measure of poverty between neighborhoods.

*Statistical analysis* - Population data to the neighborhood level was available only for the most populous municipality, Vespasiano. Despite being an urban municipality, 34 of the 44 mapped neighborhoods had a population density below 3,000 persons/km^2^, with the other ten neighborhoods having a population density averaging more than 6,500 persons/km^2^ (da Silva et al. 2013). Additionally, all but six neighborhoods were in the second quintile of purchasing power per capita in Brazil (BRL 4,100 - 15,700) with the remaining six in the fifth and sixth quintiles (BRL 27,300 - 39,000 and 39,000 - 1,029,100, respectively). None of these six higher income neighborhoods were in the high population density group. Using these data and the maps generated through ArcGIS we were able to perform a simple stratified analysis comparing neighborhoods in the same, lower, quintile of purchasing power per capita (which included 38 of 44 total neighborhoods comprising 65 cases of schistosomiasis and 46 cases of leprosy) and two different levels of population density (28 neighborhoods with lower population density and 10 with higher population density) using OpenEpi (v3.03a). The unadjusted relative risk for detecting leprosy was determined for neighborhoods with increasing numbers of cases of schistosomiasis, and average yearly incidence of leprosy was calculated for four categories of increasing incidence of schistosomiasis. For this average yearly incidence of leprosy per neighborhood comparison, two very low population neighborhoods were identified as outliers by the modified Thompson Tau method and excluded from the chart. To compare the diseases over time, incidence rates were calculated and charted for each year for both the study area as a whole and for Vespasiano. VL was included in these temporal charts to control for other temporal trends beyond those related to the association between leprosy and schistosomiasis.

*Ethics* - This study was approved by the institutional review boards of both Emory University and Faculdade da Saúde e Ecologia Humana (FASEH), Vespasiano, MG, Brazil. The procedures followed in this project are in accordance with the Helsinki Declaration of 1975, as revised in 1983.

## RESULTS

Demographic data of the three infections for all municipalities are presented in Table and include the number of cases, average age of new cases and gender. Also shown is the breakdown of MB versus PB leprosy cases.

**TABLE:** Demographic data of cases of leprosy, schistosomiasis and visceral leishmaniasis (VL) for all seven municipalities during the study period, 2007-2014

	*Mycobacterium leprae*	*Schistosoma mansoni*	VL
Total cases	139	200	315
Multibacillary, % of cases	76%	N/A	N/A
Age in years, median (range)	48 (6-97)	30 (0-84)	33 (0-89)
Gender, Male	51%	67%	61%

N/A: the multibacillary/paucibacillary classiﬁcation system does not apply to schistosomiasis or VL.

**Fig. 1:**
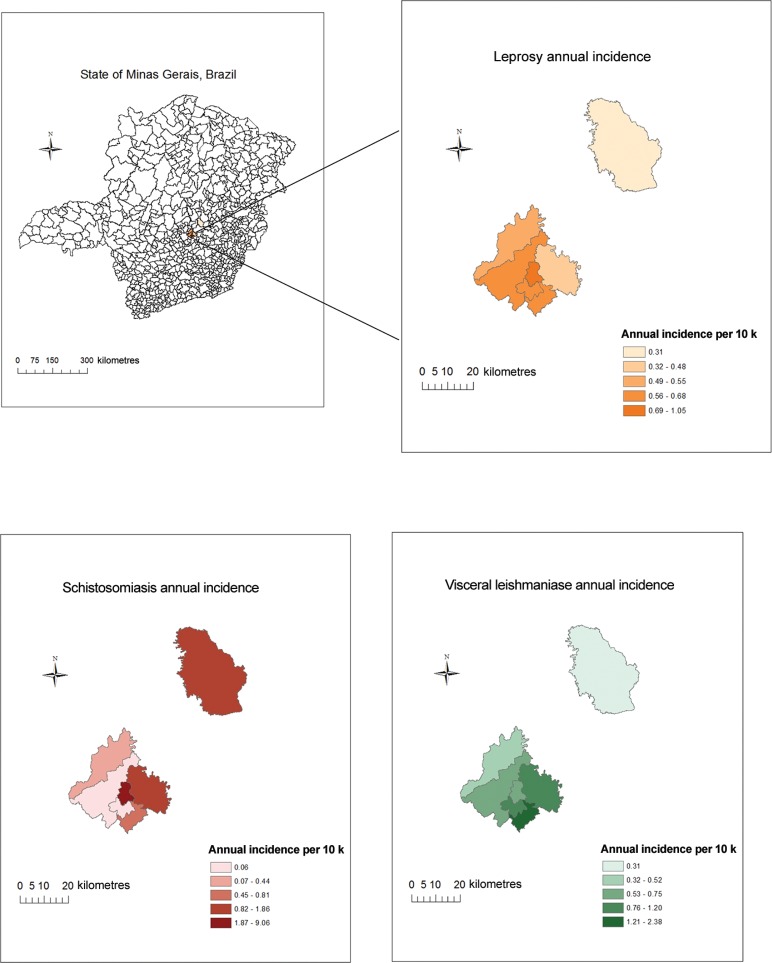
: average annual incidence for all seven municipalities of leprosy, schistosomiasis, and visceral leishmaniasis, 2007-2014.

**Fig. 2:**
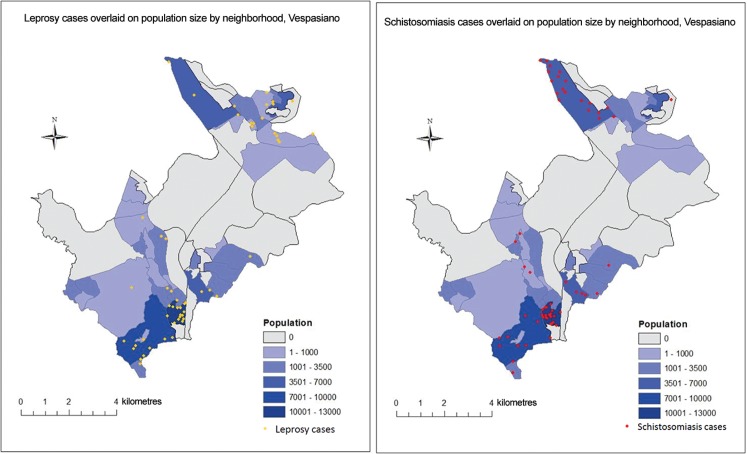
: cases per neighborhood of *Mycobacterium leprae* and *Schistosoma mansoni* infection in Vespasiano, 2007-2014. Cases on map do not represent actual addresses.

Spatial comparison of cases of leprosy, schistosomiasis, and VL in the seven municipalities studied is presented in Fig. 1. The same municipality, Confins, had the highest average incidence of leprosy (1.1/10 k) and schistosomiasis (9.3/10 k), but not of VL. Mapping at the neighborhood level for leprosy and schistosomiasis in Vespasiano is represented in Fig. 2, comparing cases of leprosy and schistosomiasis in the largest municipality juxtaposed on the population of the neighborhoods. These maps identify the similar distribution of these diseases throughout the municipality as well as areas of high burden for both infections.

**Fig. 3:**
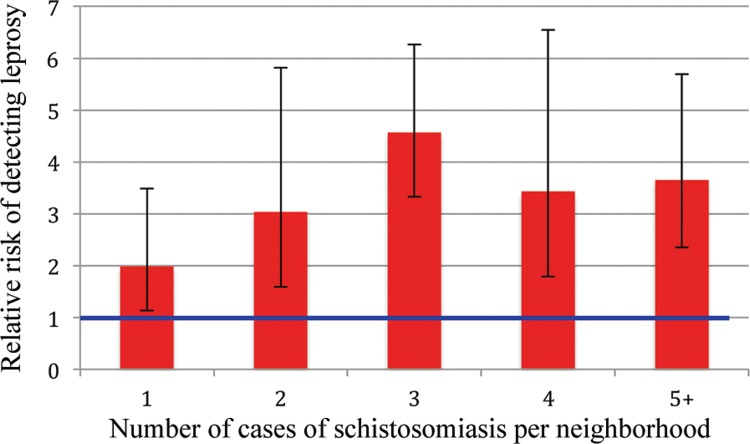
: relative risk (unadjusted) of leprosy in neighborhoods with increasing case numbers of schistosomiasis. Error bars represent 95% confidence intervals.

**Fig. 4:**
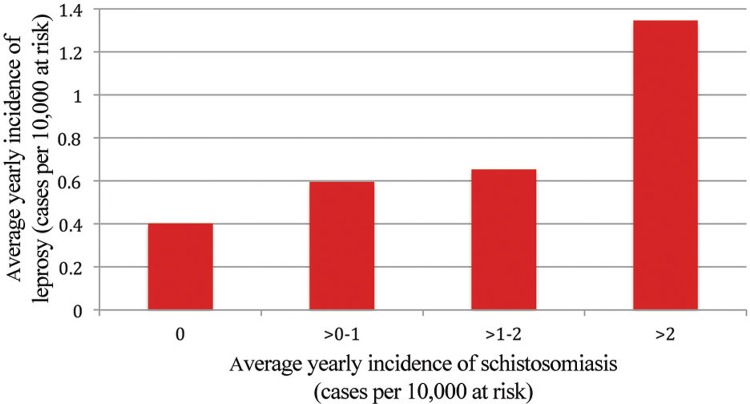
: average yearly incidence of leprosy in neighborhoods categorised by increasing average yearly incidence of schistosomiasis.

**Fig. 5:**
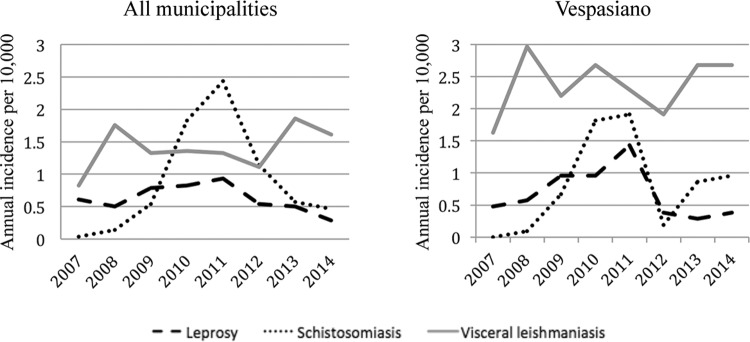
: incidence of leprosy, schistosomiasis, and visceral leishmaniasis in all seven municipalities per year from 2007-2014.

Combining these case data with the population density categories listed above and at the same purchasing power per capita quintile (38/44 neighborhoods as described above), the adjusted relative risk of leprosy in a neighborhood with reported schistosomiasis vs. those without was 6.80 [95% confidence interval (CI) 1.46 - 31.64]. The unadjusted RR before stratifying was 2.90 (95% CI 1.53-5.51). Relative risk (unadjusted) was also calculated for neighborhoods with increasing case numbers of schistosomiasis, and is presented in Fig. 3. A comparison of the average yearly incidence of leprosy vs. the average yearly incidence of schistosomiasis at different levels of schistosomiasis incidence is presented in Fig. 4. Finally, changes in incidence over time for all three diseases for the study area as a whole and for Vespasiano specifically are charted in Fig. 5. These charts show that the incidence of *S. mansoni* and *M. leprae* infections were highest for the same time period in Vespasiano (2009-2011), and for the study area as a whole (2010-2011). In both instances, both diseases peaked in 2011 before beginning to decline again. None of these trends held true for VL.

## DISCUSSION

Despite significant advances in treating and preventing *M. leprae* infection in the past three decades, leprosy continues to be a significant problem in Brazil, which carries the second highest burden of disease globally (GL 2016). Preliminary research has suggested that helminth coinfection may increase the likelihood of the most infectious form of the disease, possibly from immune dysregulation in chronic helminth infection and an increase in the Th2 response (Diniz et al. 2001, 2010). This is one potential cause of sustained transmission in places like Brazil and India, which have relatively high rates of leprosy and helminth disease in many areas (GL 2012, Couto et al. 2014, Fonseca et al. 2014, Kattula et al. 2014, Greenland et al. 2015). This project, presenting data on the first-known analysis of the geospatial and temporal overlap of leprosy and schistosomiasis in Brazil, shows a previously undescribed association between *M. leprae* and *S. mansoni* infection that can serve both to identify a need for more integrated and horizontal control efforts for these diseases as well as to form the foundation for further research into causal relationships between helminth coinfection and increased leprosy infectivity and transmission.

While this association may not be generalisable to areas without significant overlap between schistosomiasis and leprosy, it provides a case study of a potential interaction of leprosy and a helminth infection as *S. mansoni* is the predominant helminth in this area. There are areas in Brazil, however, where both infections do overlap, and given that proposed co-infection associations can and have been extrapolated to other helminths, we believe that this issue is not a major concern for the purpose of this study and associated findings.

In our analysis of Vespasiano, there is a clear association between leprosy and schistosomiasis, which is evident in the spatial overlap of the diseases and their similar temporal trends. In addition to the overlap and clustering visible on GIS-produced maps of *S. mansoni* and *M. leprae* infection, the relative risk of 6.80 (95% CI 1.46 - 31.64) of leprosy in a neighborhood with known schistosomiasis was significantly increased. With the relative risk of leprosy generally increasing with increasing neighborhood case numbers of schistosomiasis (Fig. 3), combined with the fact that average yearly incidence of leprosy increased along with incidence of schistosomiasis (Fig. 4), further supports the existence of an important link between these diseases.

Perhaps the strongest argument for the potential of a causal relationship between *S. mansoni* infection and *M. leprae* transmissibility is the fact that the relative risk of leprosy not only remained significantly increased when controlling for broad differences in income and population density, but actually increased when compared to the unadjusted value. The observed similarities in the trend of incidence for leprosy and schistosomiasis from 2007-2014, and the lack of such similarities for VL provide a final piece of evidence and a point of comparison against another endemic disease associated with many of the same traditionally understood risk factors for leprosy (poverty, lower socioeconomic status, and overcrowding) (Cabral-Miranda et al. 2014, Freitas et al. 2014).

Limitations to this study included low numbers of overall cases, which limited the extent of stratification in the analysis, the categorical nature of much of the available data (population densities, location of cases to the neighborhood level only), and limited data on other confounders such as more specific measures of poverty, crowding, and sources of infection. Some of these limitations are inherent to GIS analysis, but nevertheless limited our ability to control for additional variables and to control in a more precise fashion through more complex spatial regression analyses. There were also some inconsistencies in sources for some neighborhood boundaries and naming with some sources having more total neighborhoods than others. Regardless of the boundaries and population of any excess neighborhoods, these were all negative for both schistosomiasis and leprosy, so their inclusion would most likely have had a relatively small effect of increasing the appearance of an association. Lastly, there could be a reporting bias if not all cases of the diseases were reported to the state health department. However, for leprosy at least, reporting is mandatory in order to provide multidrug therapy for patients as it supplied by the government.

Though this project cannot prove a causal relationship between *S. mansoni* or helminth coinfection and *M. leprae* transmission, it will serve as a starting point for planned research that will address these associations further. The next phase of investigation into the question of helminths and leprosy coinfection will involve larger scale GIS studies. Georeferencing cases and higher order spatial analyses will allow for more specific comparison and greater use of the analytic tools of GIS. Direct investigations of leprosy, schistosomiasis, and other helminths in the form of co-infections will also be necessary to better delineate these associations. Combining these clinical evaluations of co-infections, including immunologic studies, with GIS epidemiologic findings will improve the body of knowledge of leprosy transmission and have the potential to truly impact disease control by increasing the tools at our disposal to reduce the reservoir of infection. These studies and results could also expand to other chronic infections of poverty that are often found in co-infections, and which have also posed significant public health challenges to elimination efforts.
